# Acupoint Sensitization, Acupuncture Analgesia, Acupuncture on Visceral Functional Disorders, and Its Mechanism

**DOI:** 10.1155/2015/171759

**Published:** 2015-08-02

**Authors:** Xiaochun Yu, Bing Zhu, Zhixiu Lin, Haifa Qiao, Jian Kong, Xinyan Gao

**Affiliations:** ^1^Institute of Acupuncture, China Academy of Chinese Medical Sciences, 16 Nanxiaojie, Dongzhimen, Beijing 100700, China; ^2^School of Chinese Medicine, Faculty of Medicine, Chinese University of Hong Kong, Shatin, New Territories, Hong Kong; ^3^Department of Biomedical Sciences, College of Medicine, Florida State University, 1115 W. Call Street, Tallahassee, FL 32306, USA; ^4^Massachusetts General Hospital, Building 120, 2nd Street, Suite 101C, Charlestown, MA, USA

Our special issue, which had opened for 6 months in the second half of 2014, focused on acupoint sensitization, acupuncture analgesia, acupuncture for visceral modulation in gastrointestinal systems, acupuncture for modulation of brain function, acupoint combination treatment of insomnia and gastrointestinal disorders, and nonspecific and specific effects of acupuncture based on stimulation intensity.

Of these papers in press, S. Chen et al. reported that the location and tenderness of Diji (SP8) were not the same in healthy subjects as in dysmenorrheal patients, suggesting dynamic and sensitivity of acupoints under different pathological status. S. Feng et al. did data mining analysis on acupoints or combinations for treatment of vascular dementia and gave suggestions for acupoint selection based on the most commonly used formulas. L. Dai et al. performed basic research in a sciatic nerve injury rat model and found that deep EA stimulation is better in improving neuromuscular function and benign regulation of apoptosis-related factors than shallow EA. J. Wang et al., based on their previous study that hippocampal mAChR-1 participating in MARK signaling was involved in EA induced cumulative analgesia in neuropathic pain rats, observed in their present study that EA2W was closely related to the cumulative analgesia via intracellular ERK and p38 MARK signaling. P. Rong et al. observed that, in anesthetized rats, EA on ST36-ST37 could enhance nucleus ventralis posterior lateralis thalami neuronal discharges which were fired by CRD-induced visceral pain. Their study indicates that acupoints may be sensitized under visceral disorders. Y. Jin et al. conducted a single blinded, randomized, controlled trial on acupuncture treatment of functional dyspepsia and found that acupuncture manipulation exhibited better effects on improving dyspeptic symptoms, mental status, and quality of life in patients with FD than nonacupoint without manipulation.

In general, we have papers involving clinical trials, data mining analysis or study protocol, and basic research in press, which thoroughly meet the expectation of our initial call for papers of this issue.

